# Transplanted ENSCs form functional connections with intestinal smooth muscle and restore colonic motility in nNOS-deficient mice

**DOI:** 10.1186/s13287-023-03469-3

**Published:** 2023-09-04

**Authors:** Ryo Hotta, Ahmed Rahman, Sukhada Bhave, Rhian Stavely, Weikang Pan, Shriya Srinivasan, Geoffrey de Couto, Luis Rodriguez-Borlado, Richard Myers, Alan J. Burns, Allan M. Goldstein

**Affiliations:** 1 Department of Pediatric Surgery, Massachusetts General Hospital, Boston, MA USA; 2 Department of Mechanical Engineering, Massachusetts Institute of Technology, Cambridge, MA USA; 3 Division of Gastroenterology, Hepatology and Endoscopy, Brigham and Women’s Hospital, Harvard Medical School, Boston, MA USA; 4 David H. Koch Institute for Integrative Cancer Research, Massachusetts Institute of Technology, Cambridge, MA USA; 5 Society of Fellows, Harvard University, Cambridge, MA USA; 6 Gastrointestinal Drug Discovery Unit, Takeda Development Center Americas, Inc., Cambridge, MA USA; 7 Stem Cells and Regenerative Medicine, UCL Great Ormond Street Institute of Child Health, London, UK

**Keywords:** Cell therapy, Enteric neuropathies, Nitric oxide synthase, Gastrointestinal motility, Optogenetics

## Abstract

**Background:**

Enteric neuropathies, which result from abnormalities of the enteric nervous system, are associated with significant morbidity and high health-care costs, but current treatments are unsatisfactory. Cell-based therapy offers an innovative approach to replace the absent or abnormal enteric neurons and thereby restore gut function.

**Methods:**

Enteric neuronal stem cells (ENSCs) were isolated from the gastrointestinal tract of *Wnt1-Cre;R26tdTomato* mice and generated neurospheres (NS). NS transplants were performed via injection into the mid-colon mesenchyme of *nNOS*^*−/−*^ mouse, a model of colonic dysmotility, using either 1 (*n* = 12) or 3 (*n* = 12) injections (30 NS per injection) targeted longitudinally 1–2 mm apart. Functional outcomes were assessed up to 6 weeks later using electromyography (EMG), electrical field stimulation (EFS), optogenetics, and by measuring colorectal motility.

**Results:**

Transplanted ENSCs formed nitrergic neurons in the *nNOS*^*−/−*^ recipient colon. Multiple injections of ENSCs resulted in a significantly larger area of coverage compared to single injection alone and were associated with a marked improvement in colonic function, demonstrated by (1) increased colonic muscle activity by EMG recording, (2) faster rectal bead expulsion, and (3) increased fecal pellet output in vivo. Organ bath studies revealed direct neuromuscular communication by optogenetic stimulation of channelrhodopsin-expressing ENSCs and restoration of smooth muscle relaxation in response to EFS.

**Conclusions:**

These results demonstrate that transplanted ENSCs can form effective neuromuscular connections and improve colonic motor function in a model of colonic dysmotility, and additionally reveal that multiple sites of cell delivery led to an improved response, paving the way for optimized clinical trial design.

## Background

Enteric neuropathies result from abnormalities of the enteric nervous system (ENS) and include conditions such as congenital aganglionosis (Hirschsprung disease), esophageal achalasia, gastroparesis, chronic intestinal pseudo-obstruction, and neurogenic constipation [1]. Broadly considered, functional gastrointestinal (GI) disorders are a major health-care burden, with over 25 billion in annual costs in the US alone [2], and enteric neuropathies are thought to represent a major cause. Despite the prevalence and severity of these diseases, current treatments show limited efficacy and often do not address the underlying pathophysiology. Cell-based therapy represents a novel approach that offers the potential to directly treat the cause of these neurointestinal diseases by replacing the absent or injured enteric neurons [3–5].

Enteric neuronal stem/progenitor cells (ENSCs) can be isolated from the GI tract of rodent [6–9] and human [10–13] or derived from human pluripotent stem cells (PSCs) [14–18]. Following transplantation into the colon of postnatal rodents, ENSCs proliferate, migrate, and form clusters of differentiated glial cells and enteric neurons [6, 7], including neuronal subtypes, with functional integration with the endogenous ENS [8]. Importantly, restoration of colonic dysmotility has been demonstrated by transplantation of ENSCs into the colon of an animal model of enteric neuropathy [9]. PSC-derived enteric neuronal progenitors improved survival in a model of Hirschsprung disease, a serious neurointestinal disorder most often seen in infants [17, 18]. While these results confirm the potential of ENSC transplantation for the treatment of enteric neuropathies, very few studies have directly shown functional neuromuscular connectivity between the transplanted ENSCs and the smooth muscle of the recipient colon. That connectivity is essential to elicit the contractile activity required to improve gut motility.

Optogenetics is a powerful tool for investigating neural function and connectivity with target organs, including in the GI tract [8, 19, 20]. Stamp et al. used optogenetics to stimulate transplanted ENSCs selectively and measured intracellular recordings from the circular smooth muscle of the recipient colon, demonstrating that graft-derived neurons functionally integrated with the muscle [8]. While this provided the first evidence of that transplant-derived neurons could elicit action potentials in smooth muscle, it did not demonstrate whether ENSCs are able to elicit contractile muscle activity. Electrical activity of the colonic smooth muscle has also been measured in isolated muscle strips ex vivo following transplantation, but not in the intact organ in vivo. Myoelectric activity is a useful indicator of neuromuscular activity in the GI tract and has been utilized as a diagnostic tool for GI neuromuscular diseases, potentially differentiating neurogenic from myogenic pathology [21]. In the present study, we combine in vivo measurement of electromyographic (EMG) activity and colorectal motility with ex vivo optogenetics and organ bath studies to provide a comprehensive characterization of the efficacy of ENSC transplantation to the colon of mice lacking neuronal nitric oxide synthase (nNOS), a model of enteric neuropathy. Our findings demonstrate that cell therapy is able to restore colonic motor function in this model and provides a strong foundation for its application in patients with neurointestinal disease.

## Methods

### Animals

This study was conducted in accordance with the protocols reviewed and approved by the Institutional Animal Care and Use Committee at Massachusetts General Hospital (Protocol #2009N000239). All methods were carried out in accordance with relevant guidelines and regulations. The reporting in the manuscript follows the recommendations in the ARRIVE guidelines.

*Wnt1::Cre* mice (Stock #003829 and Stock #009107), *R26R-tdT* reporter mice (Stock #007914), and *R26R-ChR2tdT* reporter mice (Stock #012567) were purchased from Jackson Laboratory (Bar Harbor, ME, USA). *Wnt1::Cre* mice were crossed with *R26R-tdT* and *R26R-ChR2tdT* reporter mice to generate *Wnt1::Cre;R26-tdT* (annotated as Wnt1-tdT) and *Wnt1::Cre;R26-ChR2tdT* (annotated as Wnt1-ChR2) mice, respectively.

We also generated Plp1-GFP;Wnt1-tdT mice [22] in which enteric glial cells express GFP and neural crest-derived ENS expresses tdTomato by crossing *Plp1GFP;Wnt1::Cre* mice with *R26R-tdT* mice. Plp1GFP mice [23] were kindly gifted by Dr. Wendy Macklin, University of Colorado, Denver. Heterozygote nNOS mice (Stock #002986) were purchased from Jackson Laboratory and bred to obtain homozygote nNOS knockout mice (nNOS^−/−^).

### Isolation and expansion of mouse ENSCs

ENSCs were isolated from Wnt1-tdT, Wnt1-ChR2, or Plp1-GFP;Wnt1-tdT mice as previously reported [7, 24]. Briefly, longitudinal muscle layer with myenteric plexus (LMMP) was separated from small intestine of 2–3-week-old Wnt1-tdT mice. Enzymatic dissociation was achieved using dispase (250 μg/mL; StemCell Technologies, Vancouver, BC) and collagenase XI (1 mg/mL; Sigma-Aldrich, St. Louis, MO) at 37 °C for 40 min. Single cells were isolated by filtration through a 40-µm filter and plated at 50,000 cells/mL in a 25-cm^2^ flask in mouse proliferation media, consisting of NeuroCult Mouse Basal Medium (StemCell Technologies) supplemented with 10% NeuroCult Mouse Proliferation Supplement (StemCell Technologies), 20 ng/mL epidermal growth factor (StemCell Technologies), and 10-ng/mL basic fibroblast growth factor (StemCell Technologies). After 7 days, primary neurospheres were obtained and used for transplantation experiments.

### Immunohistochemistry

Immunohistochemistry was performed on recipient mouse colon, as previously described [7, 22]. Wholemount preparations of the LMMP and enteric neurospheres were fixed in 4% paraformaldehyde. Wholemount LMMP or neurosphere preparations were permeabilized with 0.1% Triton X-100 and blocked with 10% donkey serum. Primary antibodies were diluted in 10% donkey serum and included goat anti-GFAP (1:200, Abcam, ab53554), human anti-HuC/D (Anna1, 1:16,000, kindly gifted by Lennon laboratory) mouse anti-HuC/D (1:50, Invitrogen, A-21271), rabbit anti-nNOS (1:100, Cell Signaling, C7D7), rabbit anti-Sox10 (1:400, Abcam, ab155279), and mouse anti-neuronal class III conjugated β-tubulin (Tuj1; 1:400; Covance, Dedham, USA). Secondary antibodies included anti-rabbit IgG (1:500; Alexa Fluor 488; Fisher Scientific Life Technologies) and anti-human IgG (1:200, Alexa Fluor 594; Fisher Scientific Life Technologies). Cell nuclei were stained with DAPI (Vector Labs, Burlingame, CA) and mounted with aqua-poly/mount (Fisher Scientific Polysciences Inc.). Images were taken using Nikon A1R laser scanning confocal microscope (Nikon Instruments, Melville, NY) or Keyence BZX-700 All-In-One Microscopy system (Keyence America, Itasca, IL).

### Cell delivery to mouse colon in vivo

**Fig. 1:**
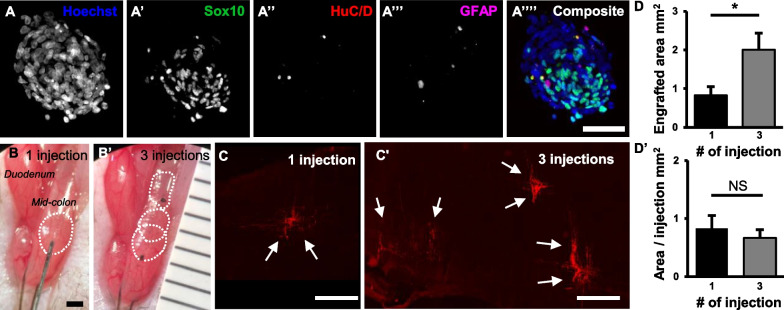
Multiple injections of ENSCs to colon of *nNOS*^*−/−*^ mouse increase cell coverage. Immunohistochemical characterization of ENSCs (**A**) and their transplantation to mouse mid-colon in vivo (**B**). Six weeks following cell injection, wholemount preparation was used to measure the area covered by transplanted ENSCs (**C**). Multiple injections covered significantly larger area than single injection (**D**, *p < 0.05). Scale bars 50 µm (**A′**–**A′″**) and 1 mm (**B** and **C**)

Eight- to twelve-week-old mice were used as recipient mice for transplant study. Recipient mice were anesthetized by isoflurane inhalation. A midline abdominal skin incision was made, and the mid-colon exposed. Cell suspension was prepared at 10 neurospheres per μL or 30 neurospheres per μL, and 3 μL injected per site as summarized in Table 1. A 3-μL cell suspension was distributed along 2-mm length of the anterior wall of the mid-colon (Fig. 1B). For three injections, sites of injection were oriented longitudinally along the antimesenteric side of the colon (Fig. 1B′). After cell injection, sites were tattooed with India ink for later identification (Fig. 1B′).

**Table 1:** Experimental groups tested in this study

Groups	Volume per injection (μL)	NS concentration (NS per μL)	# of NS per injection	# of injections	# of NS per animal
30 NS × 1 injection	3	10	30	1	30
30 NS × 3 injections	3	10	30	3	90
90 NS × 1 injection	3	30	90	1	90

*NS* neurospheres

### Assessment of colorectal motility in vivo

Functional outcomes on GI motility following cell transplantation were assessed by measuring rectal bead expulsion time, counting the number of fecal pellet output, and electromyography.

1. *Rectal bead expulsion (RBE) time* A glass bead 3 mm in diameter was inserted 1 cm into the distal colon using a silicone pusher and the time to bead expulsion determined with time limit set at 100 min [25].
2. *Fecal pellet output (FPO)* A single mouse was housed in an individual cage without bedding 30 min before starting the analysis. Animals had free access to food and water. Fecal pellets expelled were collected every 30 min, calculating the total number of expelled pellets for 2-h period [19, 26].
3. In vivo* electrophysiology* Electrophysiological metrics were used to assess the efficacy of the cell transplantation methodology. Luminal recordings were carried out on a RHS Stimulation/Recording System (Intan Technologies) at 30-kHz sampling frequency. All procedures were performed under the approval of the institutional review board (IRB) of Massachusetts General Hospital (MGH). Mice of either sex and their wild-type control littermates (Jackson Laboratory, Maine, USA) were anesthetized using 1–5% isoflurane. Following anal dilation, an enema was performed using 2-4 mL of saline. Four to six evenly spaced bipolar electrodes situated on a custom probe connected to a 32-pin header (Omnetics) were inserted into the distal colon, and electrical activity was recorded for at least 20 min. Upon euthanasia, a laparotomy was performed, and the tissue was inspected for perforation and correct placement of the electrodes.

### Organ bath smooth muscle activity study

After collecting in vivo data, colonic smooth muscle function was further analyzed ex vivo using standard organ bath techniques as described previously [22]. Freshly excised distal colon was quickly placed in a Petri dish containing physiological Krebs’ solution (118 mmol/L NaCl, 4.7 mmol/L KCl, 1.2 mmol/L MgSO_4_·7H_2_O, 1.2 mmol/L KH_2_PO_4_, 25 mmol/L NaHCO_3_, 11.7 mmol/L glucose, and 1.25 mmol/L CaCl_2_, all chemicals from Sigma-Aldrich, St. Louis, MO, USA). Colonic segment marked by Indian ink was cut into a 5-mm ring. The colonic rings were then mounted between two small metal hooks attached to force displacement transducers in a muscle strip myograph bath (Model 820 MS; Danish Myo Technology, Aarhus, Denmark) containing 7 mL of physiological Krebs’ solution (oxygenated with 95% O_2_ and 5% CO_2_) maintained at 37 °C. Then, the rings were stretched to give a basal tension of 0.5 g and were equilibrated for 60 min in Krebs’ solution changed every 20 min. Force contraction of the circular smooth muscle was recorded and analyzed by using a Power Lab 16/35 data acquisition system (ADInstruments, NSW, Australia) and a computer via Lab Chart Pro Software v8.1.16 (ADInstruments). Electrical field stimulation (EFS) was applied via stimulation electrodes (built into the chamber cover) connected to the CS4 constant voltage stimulator (Danish Myo Technology, Aarhus, Denmark). Colon segments were stimulated for 15 s, and the stimulation frequency (5 Hz), voltage (40–60 V), and pulse duration (300 μs) were controlled by MyoPulse software (Danish Myo Technology, Aarhus, Denmark). EFS was applied to the bath in non-adrenergic non-cholinergic conditions (atropine; 1 mM, phentolamine hydrochloride; 1 mM, and propranolol hydrochloride; 1 mM). EFS was also applied 5 min before and after nitric oxide synthase antagonist, L-NAME (100 mM) was added to the bath solution to examine the nitrergic-mediated responses. Baseline values were obtained by averaging 60 s of data 5 min prior to EFS, and maximum changes for rebound contraction (Phase-II) were expressed as absolute or percentage changes from baseline values. The area under the curve (AUC), less baseline, during first 10 s of EFS period was determined as Phase-I (relaxation). To assess the viability and confirm the functionality of the tissues, contractile responses to 60-mM KCl were performed and compared at the beginning and at the end of each experiment.

### Optogenetics stimulation of the smooth muscle

Segment of recipient colon was prepared as above. Blue light stimulation (BLS) was applied using a diode-pumped solid-state laser system (470 nm, 200 mW, Model number: MDL-III-470; OptoEngine, LLC, Midvale, UT). Trains of light pulses (20-ms pulse width, 10 Hz, 15-s train duration) were shone focally on the serosal surface of the transplanted colon in the organ bath via a glass fiber optic (200-μm diameter).

### Electrophysiological analysis

Data from the Intan system were exported and processed in MATLAB. Data were bandpass and notch filtered (60 Hz) to remove low-frequency motion artifact, breathing artifact, and white noise. The data were further rectified and normalized based on the mean rectified signal. The contractile rate was defined by the number of peaks occurring per unit time. A dormant section of data was manually selected to represent a baseline resting condition. The mean value of this data was designated to be the baseline resting amplitude. Peaks were defined as clusters of spikes at least 1.5 times greater than the baseline resting amplitude and at least 10 ms from the nearest neighbor to prevent double counting the same spike.

### Statistical analysis

Data analysis was performed using GraphPad Prism v8 (GraphPad Software, Inc., San Diego, CA). Two-tailed *t*-tests were performed for pairwise comparisons. A one-way analysis of variance (ANOVA) was performed with a post hoc Holm–Sidak test for multiple comparisons. For all analyses, *p* < 0.05 was considered significant. All data were presented as mean ± SEM, unless otherwise stated.

## Results

### Isolation, expansion, and transplantation of mouse ENSCs to mid-colon of nNOS^−/−^ mouse in vivo

Mouse ENSCs were isolated and expanded in culture to form neurospheres (NS) (Fig. 1A–A″″). Immunohistochemical characterization confirmed the presence of Sox10-expressing neural progenitors (Fig. 1A′), HuC/D + neurons (Fig. 1A″), and GFAP + glial cells (Fig. 1A′″). A 3-ul suspension of NS was delivered via microinjection into the anterior wall of the mid-colon of 3-month-old *nNOS*^*−/−*^ mice (*n* = 12, Fig. 1B, dotted circle). Cell injections were performed in three groups (Table 1): Group 1 (30 NS × 1 injection site, *n* = 12, Fig. 1B); Group 2 (30 NS × 3 injection sites positioned longitudinally along the antimesenteric colon, *n* = 12, Fig. 1B′); and Group 3 (90 NS × 1 injection site, *n* = 5). Injection sites were tattooed with India ink for later identification (Fig. 1B′). Six weeks following cell transplantation, wholemount preparation of recipient colon showed successful engraftment of transplanted ENSCs (Fig. 1C, arrows). The area covered by transplanted ENSC-derived cells and fibers was significantly larger in the three-injection group as compared to a single injection (Fig. 1C′ and D; 2.01 ± 0.55 mm^2^ vs. 0.84 ± 0.29 mm^2^, *n* = 3, *p* < 0.05), while the area covered by each individual injection was not significantly different (Fig. 1D′).

### Transplanted ENSCs migrate, extend neuronal fibers, and give rise to enteric glial cells and neurons

**Fig. 2:**
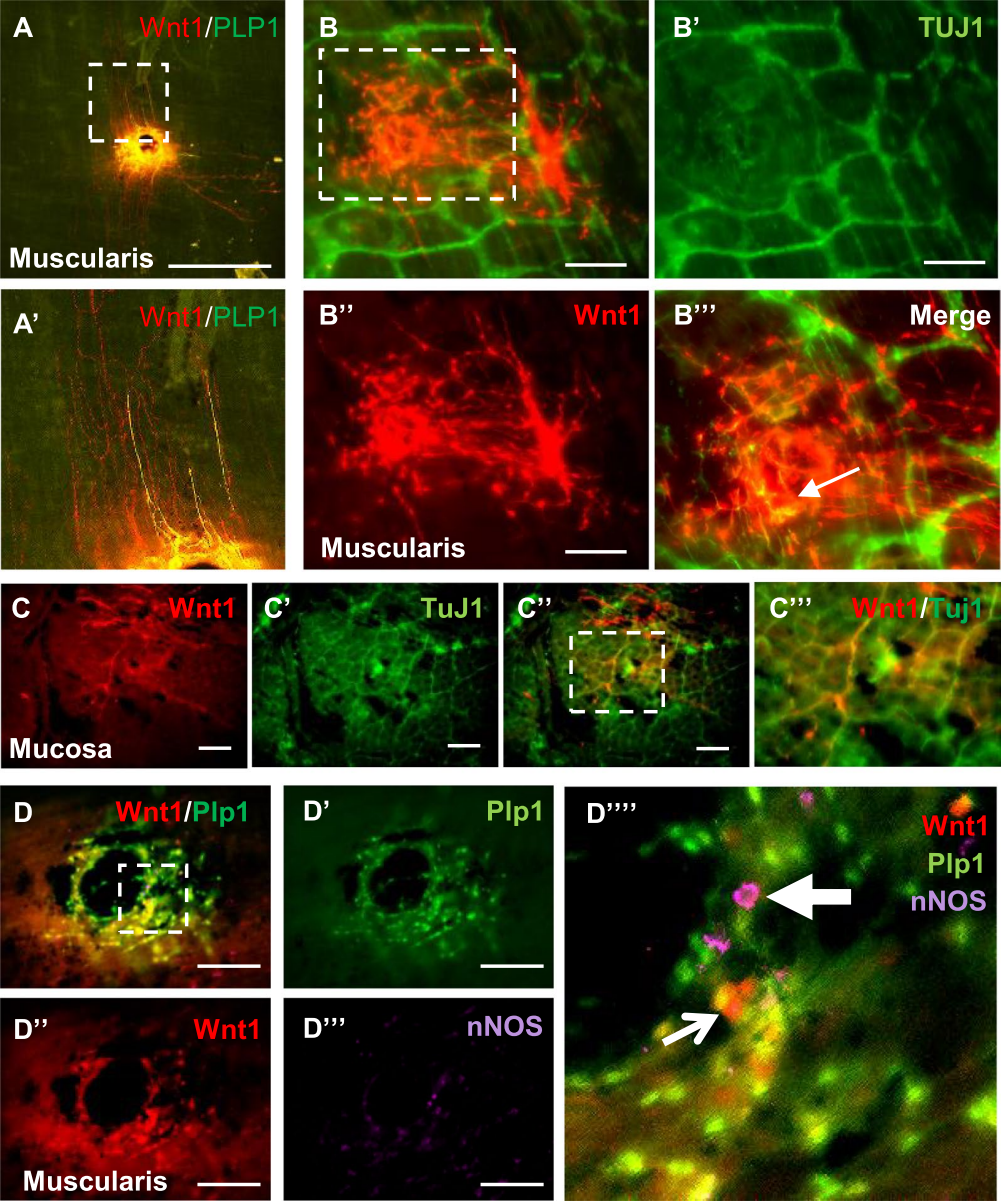
ENSCs give rise to glial cells and neurons, including nNOS-expressing subtype, 6 weeks following transplantation to mouse colon in vivo. Transplanted ENSCs were identified as tdT + cells and fibers, with co-localization with the glial marker, Plp1 in the muscularis layer (**A**) while some projecting fibers derived from transplanted cells are Plp1 negative (**A′**, a magnified view of dotted box shown in **A**). Wholemount immunostaining with Tuj1 (**B**–**B′″**) shows neuronal differentiation of transplanted ENSCs (**B′″**, arrow, **B′″** is a magnified view of dotted box shown in **B**). In the mucosal layer (**C**–**C″**), transplanted ENSC-derived neuronal fibers are integrated with host neurons (**C′″** is magnified view of dotted box shown in **C″**). A subpopulation of ENSC-derived neurons express nNOS (**D**–**D″″**, arrow, **D′″** is magnified view of dotted box shown in **D**). Scale bars 1 mm (**A**) and 200 µm (**B**–**D**)

Immunohistochemical examination of wholemount preparation obtained from recipient *nNOS*^*−/−*^ mouse colon showed engraftment of Wnt1-tdT + transplanted cells with co-expression of the glial marker, Plp1 (Fig. 2A, magnified in Fig. 2A′). Staining with the neural marker Tuj1 (Fig. 2B–B″) showed neuronal differentiation of transplanted Wnt1-tdT + cells (Fig. 2B′″, arrow). Neuronal fibers extending from the transplanted cells appeared to integrate with host endogenous neuronal fibers within the submucosal layer (Fig. 2C–C″″). Furthermore, subpopulations of Wnt1-tdT + transplanted cells were immunoreactive for nNOS (Fig. 2D–D″″, arrows), suggesting their differentiation into nitrergic neurons, which are missing in the recipient nNOS-deficient colon.

### Transplantation of mouse ENSCs partially restores colorectal motility in nNOS^*−/−*^ mice

**Fig. 3:**
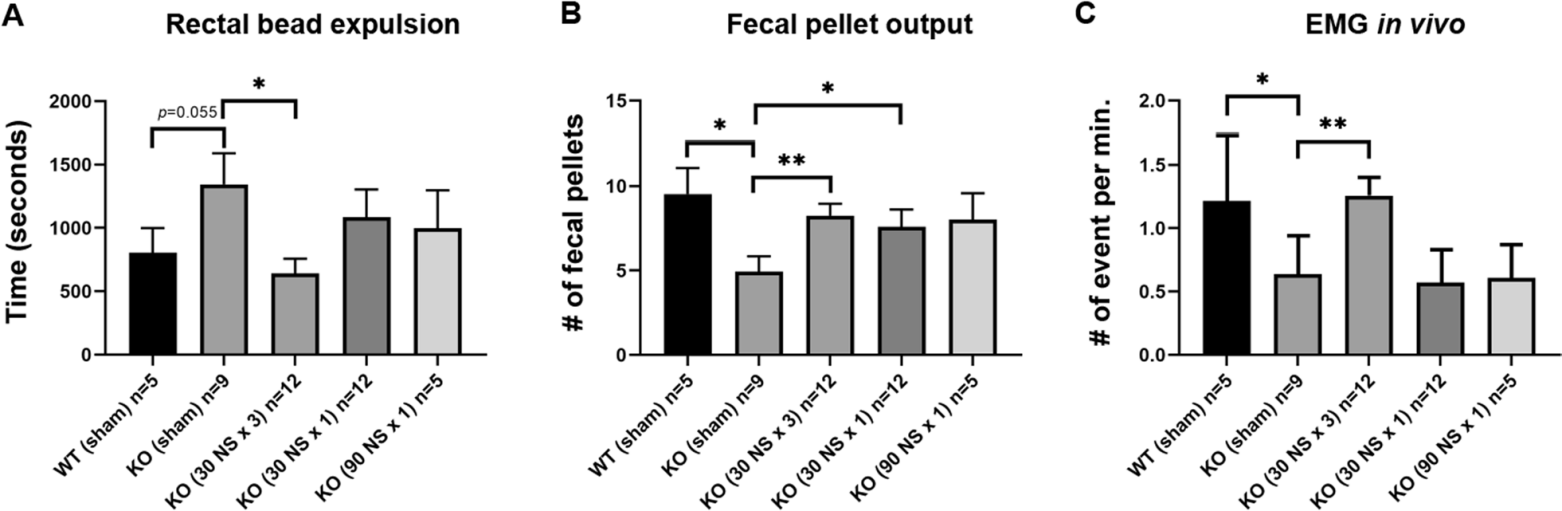
Multiple injections of ENSCs partially restore colorectal dysmotility of nNOS^*−/−*^ mouse in vivo. Delayed rectal bead expulsion time seen in Sham-operated nNOS^*−/−*^ (KO) mice is normalized by multiple injections of ENSCs [**A** KO (Sham) vs. KO (30NS × 3), *p < 0.05], but not by single injection. Fecal pellet output is significantly lower in Sham-operated KO (**B** *p < 0.05) whereas injection of ENSCs restored this phenotype [**B** KO (Sham) vs. KO (30NS × 3), **p < 0.01; KO (Sham) vs. KO (30NS × 1), *p < 0.05]. Colonic smooth muscle activity measured by electromyography in vivo demonstrated significantly decreased activity in Sham-operated KO in comparison to Sham-operated WT [**C** WT (Sham) vs. KO (Sham), *p < 0.05] whereas multiple injections of ENSCs significantly improved muscle activity [**C** KO (Sham) vs. KO (30NS × 3), **p < 0.01]

Colorectal function of recipient mice was assessed 6 weeks following cell transplantation. nNOS-deficient mice are known to have upper and lower GI dysmotility [9, 27]. Following cell therapy, however, we observed a marked improvement in colorectal motility. Rectal bead expulsion time was longer in *nNOS*^*−/−*^ Sham group compared to WT littermates that underwent Sham surgery (Fig. 3A, *p* = 0.055). Cell transplantation with three injections of 30 NS each significantly reduced rectal bead expulsion time in *nNOS*^*−/−*^ mice (Fig. 3A, KO (Sham) vs. KO (30 NS × 3), *p < 0.05). In contrast, a single injection of 30 NS did not improve expulsion time (Fig. 3A). We examined the number of fecal pellets expelled over 2 h to evaluate colorectal motility. Sham-operated *nNOS*^*−/−*^ mice expelled significantly fewer pellets in comparison with the WT Sham group (Fig. 3B, WT (Sham) vs. KO (Sham), *p < 0.05), and ENSC transplantation recovered this phenotype (Fig. 3B, KO (Sham) vs. KO (30 NS × 3), **p < 0.01; and KO (Sham) vs. KO (30 NS × 1), *p < 0.05).

The contractile rate, defined by the number of bursting clusters per minute, among KO mice was significantly lower as compared to WT based on electromyographic (EMG) recording. Periodic stimulation was performed to evaluate the colon’s response to excitation (10 Hz, 50 μA, three 10-μs pulses 90 s) in vivo with the animals under general anesthesia. In these conditions, the contractile rate of the KO group was significantly lower than WT (Fig. 3C, WT (Sham) vs. KO (Sham), *p < 0.05). Cell transplantation with three injections resulted in a significantly increased contractile rate in KO mice (Fig. 3C, KO (Sham) versus KO (30 NS × 3), **p < 0.01), again confirming ENSC-mediated restoration of colorectal motility in *nNOS*^*−/−*^ mice. Finally, to determine whether these effects are derived from the increased number of injections or from the increased number of cells administered, we delivered 90 NS to the mid-colon of *nNOS*^*−/−*^ mice by a single injection (90 NS × 1 in Fig. 3A–C). This, however, did not improve colonic motility by any of the parameters measured (Fig. 3A–C). Consequently, further experiments did not include testing of a single injection of 90 NS.

### Transplantation of mouse ENSCs restores colonic smooth muscle relaxation in nNOS^*−/−*^ mice

**Fig. 4:**
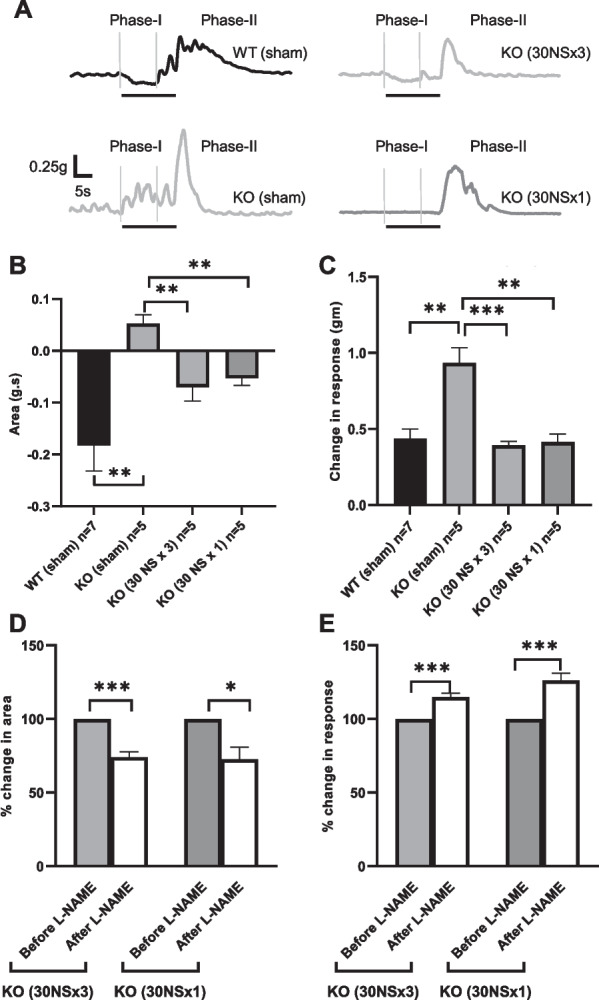
Restoration of circular smooth muscle activity in *nNOS*^*−/−*^ (KO) colon by transplantation of ENSC. **A** Representative traces of mechanical response to EFS in NANC conditions. EFS induced a biphasic response characterized by a relaxation (Phase-I) followed by a rebound contraction (Phase-II). **B** and **C** Quantification of EFS-induced relaxation (area under curve) and rebound contraction (maximum effects are shown as absolute change from basal values). **D** and **E** Group data illustrate the effects of L-NAME on EFS-induced muscle activity in transplanted mice. Error bars represent mean ± SEM. Numbers of animals are shown in parentheses. ***p < 0.001, **p < 0.01, and *p < 0.05

To determine if ENSC transplantation can restore the nitrergic response in nNOS-deficient mice, organ bath studies were performed. In non-adrenergic non-cholinergic (NANC) conditions, electrical field stimulation (EFS) induced a prolonged relaxation (Fig. 4A, WT (Sham), Phase-I), followed by a post-stimulation rebound contraction (Fig. 4A, WT (Sham), Phase-II) in colonic smooth muscle from WT mice. In contrast, smooth muscle obtained from *nNOS*^*−/−*^ mice exhibited no muscle relaxation in response to EFS (Fig. 4A, KO (Sham), Phase-I) and an exaggerated post-stimulation rebound contraction (Fig. 4A, KO (Sham), Phase-II). Six weeks following ENSC transplantation, *nNOS*^*−/−*^ mice demonstrated restoration of the inhibitory response (Fig. 4A, KO (30 NS × 3) and KO (30 NS × 1), Phase-I). The lack of relaxation in *nNOS*^*−/−*^ smooth muscle (Fig. 4B, − 0.18 ± 0.05 g s in WT (Sham) vs. 0.05 ± 0.02 g.s in KO (Sham), **p < 0.01) was rescued by ENSC transplantation (Fig. 4B, 0.05 ± 0.02 g s in KO (Sham) vs. − 0.07 ± 0.03 g s in KO (30 NS × 3) or − 0.05 ± 0.01 in KO (30 NS × 1), respectively, **p < 0.01). Moreover, the exaggerated post-stimulation contraction observed in *nNOS*^*−/−*^ mice (Fig. 4C, 0.44 ± 0.06 g in WT (Sham) vs. 0.93 ± 0.10 g in KO (Sham), **p < 0.01) was eliminated by ENSC transplantation (Fig. 4C, 0.39 ± 0.03 g in KO (30 NS × 3) and 0.41 ± 0.05 g in KO (30 NS × 1), **p < 0.01).

To confirm that the restored relaxation response was due to the transplanted ENSC-derived nitrergic neurons, EFS was performed in the presence and absence of the nitric oxide synthase (NOS) blocker L-NAME in NANC conditions. L-NAME significantly reduced EFS-induced relaxation by 26% following three injections (*p* < 0.001, *n* = 5) and by 28% following one injection (*p* < 0.05, *n* = 5) (Fig. 4D). Similarly, post-stimulation rebound contraction was significantly increased after L-NAME by 15% (*p* < 0.001, *n* = 5) and 26% (*p* < 0.001, *n* = 5), respectively (Fig. 4E), confirming that these effects were mediated by nitric oxide.

### Optogenetic activation of transplanted ENSCs leads to colonic smooth muscle contractility in recipient mice

**Fig. 5:**
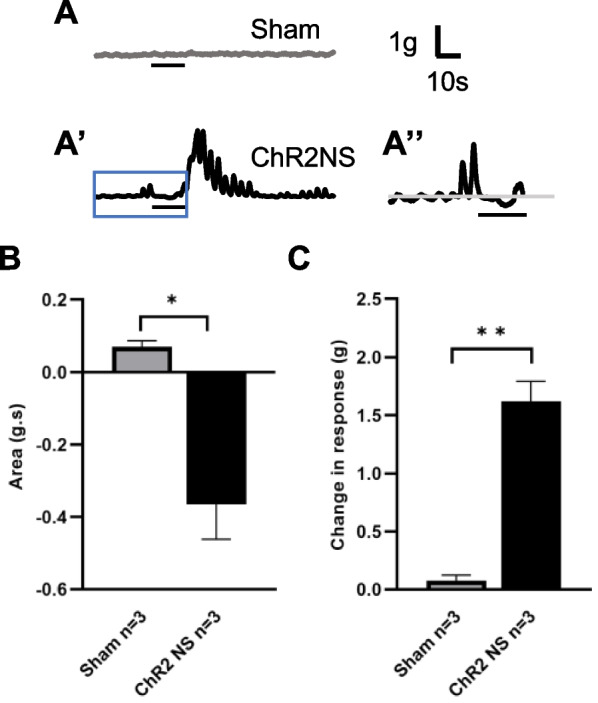
Restoration of circular smooth muscle activity in *nNOS*^*−/−*^ mouse colon by transplantation of ENSC-ChR2 cells. **A**–**A″** Representative traces of mechanical response of colonic smooth muscle to BLS. Sham-operated *nNOS*^*−/−*^ colon showed no response to BLS **A** whereas BLS induced a biphasic response characterized by initial relaxation followed by a rebound contraction in mice with transplantation of ChR2 NS (**A′**–**A″**, **A″** is magnified view of blue box shown in **A′**). **B** and **C** Quantification of BLS-induced relaxation (area under curve) and rebound contraction (maximum effects are shown as absolute change from basal values). Error bars represent mean ± SEM. Numbers of animals are shown in parentheses. **p < 0.01 and *p < 0.05

To confirm whether EFS-induced responses were due directly to the neuronal activation of transplant-derived cells, we isolated ENSCs from 3-week-old *Wnt1::Cre;R26-ChR2* (Wnt1-ChR2) mice, in which all neural crest-derived cells express the light sensitive ion channel, channelrhodopsin-2 (ChR2), and transplanted these cells to *nNOS*^*−/−*^ mouse colon in vivo. Sham surgery consisted of transplanting ENSCs isolated from Wnt1-tdT (ChR2-negative) mice. Three weeks later, the transplant site was harvested, and organ bath studies were performed. Colonic smooth muscle from *nNOS*^*−/−*^ mice displayed no relaxation, quiescence, or rebound contraction in response to blue light stimulation (BLS) (Fig. 5A, Sham). In contrast, BLS induced a relaxation response followed by a rebound contraction in colons that received Wnt1-ChR2 cells (Fig. 5A′, ChR2NS and Fig. 5A″, magnified view of blue box shown in A′). Quantitative comparison demonstrated both a significant inhibitory (Fig. 5B, 0.07 ± 0.02 g s in Sham vs. − 0.36 ± 0.10 g s in ChR2 NS, *p < 0.05) and excitatory (Fig. 5C, 0.07 ± 0.05 g in Sham vs. 1.62 ± 0.17 g in ChR NS, **p < 0.01) response. These results show that activation of transplanted neurons produces measurable muscle activity, confirming effective neuromuscular connectivity of the transplanted ENSCs.

## Discussion

Regenerative cell-based strategies to restore function in neurointestinal diseases requires that transplanted neurons establish effective neuromuscular connections with intestinal smooth muscle. Prior studies have demonstrated successful neuromuscular communication in cultured neurons and smooth muscle cells [17] and functional integration between transplanted ENSC-derived neurons and the host ENS [8]. However, those studies have not shown that transplanted ENSCs can form neuromuscular integration that elicits myoelectrical activity and results in smooth muscle contractility in vivo. In the current study, we isolated and expanded ENSCs as neurospheres and transplanted them into the colon of *nNOS*^*−/−*^ mouse, which have colonic dysmotility, to test whether transplanted ENSCs can form functional neuromuscular connections with smooth muscle in the recipient colon and lead to improved colonic motility. Using multiple electrophysiologic assays, including EFS, EMG, and optogenetics, we found that transplanting ENSCs into a broader area resulted in greater cell and neurofiber coverage, functional integration with the host smooth muscle, and a significant improvement in colonic motor function. This study highlights the clinical potential of cell therapy for neurointestinal diseases and demonstrates a feasible approach to cell delivery that enhances functional efficacy. Moreover, this is the first study to demonstrate directly, using optogenetics, the functional integration of transplanted cells with the colonic smooth muscle.

The ENS is comprised of glial cells and neurons that are classified into at least 15 subtypes based on their function and chemical coding [28, 29]. Neuronal nitric oxide synthase (nNOS) catalyzes the production of nitric oxide (NO), which functions as an inhibitory neurotransmitter in the GI tract and thus relaxes the smooth muscle. Loss or disruption of nNOS neurons occurs in a range of human enteric neuropathies [30, 31], including esophageal achalasia [32–34], GI dysmotility associated with diabetes [35–37], and Parkinson’s disease [38]. Cell therapy offers an approach to replacing missing or damaged neurons in the GI tract to improve gut motility [3–5]. We and others have shown improvement in upper and lower GI motility following ENSC transplantation in nNOS-deficient mice [9, 27]. Consistent with the previous studies in nNOS-deficient mice, we show that ENSCs can differentiate into nNOS-expressing neurons, effectively restore inhibitory neurotransmission, and reverse the associated colorectal dysmotility in these mice [9].

Although neuronal cell therapy holds potential to treat enteric neuropathies by directly addressing the underlying pathophysiology, several potential concerns need to be taken into consideration, including long-term safety and efficacy. One study has described the successful long-term engraftment of transplanted ENSCs within the mouse colon for up to 2 years [39]. Cooper et al. transplanted mouse-derived ENSCs into the colon of wild-type mice, where neuroglial differentiation and neurofiber projections from transplanted ENSCs were observed 2 years post-surgery without evidence of tumor formation or spread to other organs [39]. They also demonstrated that transplanted ENSC-derived neurons integrated into the host ENS using live cell calcium imaging. However, they did not examine whether the smooth muscle is able to contract in response to activation of the transplanted cells, which we have shown in the current study. Unfortunately, that study was unable to test the functional contribution of the transplanted ENSCs to intestinal motility since the recipient animals were wild type and, therefore, had no deficit in colonic function. Regarding the risk for tumor formation, this will be a particularly important concern if pluripotent stem cells (PSCs) are used as opposed to tissue-derived ENSCs [3].

Two previous studies have demonstrated the potential of PSCs to treat enteric neuropathies [17, 18]. One group successfully induced enteric neural progenitors (ENPs) from PSCs and transplanted them to the colon of mice with Hirschsprung disease, a congenital ENS disorder associated with a severe and lethal phenotype. They showed that transplantation of PSC-derived ENPs restored the colonic migrating motor complex 4 weeks after surgery and led to prolonged survival of these mice [17, 18]. These authors followed the recipient mice up to 9 months and found no evidence of tumor formation in the recipient gut. Therefore, while studies on long-term efficacy and safety after cell therapy for neurointestinal diseases are limited, our use of a non-lethal animal model of enteric neuropathy (nNOS-null mice) with functional readouts that do not require euthanasia (in vivo EMG and GI motility assays) will allow us to answer these critically important questions of translational relevance for future clinical application.

Some of the major challenges in ENSC transplantation have been the limited migratory capacity and poor survival of the transplanted cells, particularly when using postnatal-derived ENSCs. It has been repeatedly shown that postnatal tissue-derived stem/progenitor cells, including ENSCs, demonstrate significantly lower ability to migrate compared to their embryonic counterparts, but the ethical and immunological hurdles of using the former are considerably lower [3, 40]. Attempts to overcome these difficulties have included engineering the cells prior to transplantation [41–43] or co-delivering neurotrophic growth factors [44]. We previously shown that co-injection of ENSCs with serotonin agonist-loaded nanoparticles can optimize survival and neuronal differentiation of ENSCs following transplantation into the mouse colon in vivo [45]. No previous study has attempted to improve functional outcome by optimizing the area of cell coverage. Our multiple injections method improved cell coverage and was associated with enhanced functional recovery, supporting for the first time a relationship between the area of transplant cell engraftment and functional improvement. McCann et al. showed that a single implantation of 3 NS (~ 6 × 10^4^ cells in total) was enough to reverse the colonic dysmotility of nNOS-null mice [9]. They observed that ENSCs were capable of migrating out from the implanted neurospheres and covered up to 5 mm^2^ of the gut wall 2 weeks following transplantation [9]. They also reported an important contribution of the interstitial cells of Cajal and a paracrine effect of transplanted ENSCs on the restoration of GI dysmotility. It is not clear if a certain area of cell coverage is required to restore gut motility, but increasing the number of injection/implantation sites to maximize the coverage area is a feasible and effective approach to elicit a functional benefit.

This is the first study utilizing in vivo electromyographic recording of the colonic smooth muscle to demonstrate that ENSC transplantation can restore myoelectrical activity in mice with an enteric neuropathy. Studies on the physiology of the GI tract date back almost 60 years, with the development of systems to study GI electromechanical activity beginning in the 1970s [46]. Myoelectric activity of intestinal muscles has been recorded with electrodes placed on the serosal or mucosal surface of the intestine in laboratory animals [47–50] and humans to study electrical activity in health and disease [51, 52]. Refinements allowed for removal of electrocardiogram interference and engineering of more sensitive and finer electrodes, with subsequent development of noninvasive surface electroenterography using cutaneous electrodes [53, 54]. In recent years, research on cutaneous high-resolution mapping of gastric slow waves using array matrix electrodes has revealed new features of gastric myoelectric dysfunction that may contribute to our understanding of functional GI disorders [55, 56]. However, little progress has been made in measuring myoelectrical colonic activity less invasively. In the present study, a novel electrophysiological recording system was utilized to measure electrical activity in the colon of mice with enteric neuropathy. Interestingly, significantly reduced electrical activity was seen in mice lacking the inhibitory neurotransmitter, nNOS, and this was reversed by ENSC therapy. Although further investigations will be required to understand the mechanisms underlying this phenomenon, this new device adds value to cell transplant studies, allowing functional analysis of ENSC transplants over time without requiring euthanasia of the recipient animals.

While it has been shown that the inhibitory response in *nNOS*^*−/−*^ mouse colon can be restored by transplantation of ENSCs [9], it was not previously clear if the transplant-derived enteric neurons were solely responsible for this inhibitory effect because direct relaxation of intestinal smooth muscle by EFS has been described [57]. Stamp et al. demonstrated functional integration of transplanted ENSCs with the endogenous ENS by performing intracellular electrical recordings of endogenous colonic myenteric neurons while activating transplanted ENSCs-expressing ChR2 [8]. In the present study, we transplanted ChR2-expressing ENSCs to nNOS-null mouse colon and confirmed smooth muscle contraction in response to light activation of those cells. We and others have observed that functional connectivity between transplanted ENSCs and endogenous ENS [8] or intestinal smooth muscle can be detected using organ bath studies as early as 2–3 weeks post-transplant [9, 22, 58]. Therefore, we performed the organ bath experiments at 3 weeks following cell injection and examined colonic motility in vivo at 6 weeks post-surgery since we find that those additional 3 weeks are beneficial for the transplanted ENSCs to elicit a more significant improvement in colonic motor function (RH unpublished observation).

One limitation of our study is that we cannot be certain which cell type was responsible for the effects we observed since we generated ENSCs from Wnt1-ChR2 mice, in which all neural crest-derived cells express ChR2, including all enteric neuronal subtypes and glial cells. In addition, we did not assess the specific molecular pathways involved in the improved response of colonic smooth muscle to ENSC activation. Presumably, this involves stimulation of both cholinergic (excitatory) and nitrergic (inhibitory) enteric neuronal signaling, thereby leading to improved motor function, but the detailed mechanisms still need to be worked out. Nevertheless, our results confirm that transplanted ENSCs can regulate colonic motor activity, an important step forward in the development of cell therapy to treat neurointestinal disease.

## Conclusions

In summary, we have shown that maximizing cell coverage using multiple ENSC injections leads to improved colonic motor function in nNOS-deficient mice, a model of enteric neuropathy. Optogenetics and electrophysiologic studies demonstrate that transplanted ENSCs form functional neuromuscular connections that improve colorectal motility. These findings represent an important step toward the design of first-in-human clinical trials.

## Data Availability

The datasets used and/or analyzed during the current study available from the corresponding author on reasonable request.

## Abbreviations

BLS: Blue light stimulation
ChR2: Channelrhodopsin-2
EFS: Electrical field stimulation
ENS: Enteric nervous system
ENSC: Enteric neuronal stem cells
EMG: Electromyography
FPO: Fecal pellet output
GI: Gastrointestinal
LMMP: Longitudinal muscle layer with myenteric plexus
nNOS: Neuronal nitric oxide synthase
NS: Neurospheres
PSC: Pluripotent stem cells
RBE: Rectal beads expulsion
